# Multiple Emission Peaks Challenge Polariton Condensation in Phenethylammonium-Based 2D Perovskite Microcavities

**DOI:** 10.1021/acsphotonics.4c02065

**Published:** 2025-04-17

**Authors:** Martin Gomez-Dominguez, Victoria Quirós-Cordero, Esteban Rojas-Gatjens, Katherine A. Koch, Evan J. Kumar, Carlo A. R. Perini, Natalie Stingelin, Carlos Silva-Acuña, Ajay Ram Srimath Kandada, Vinod Menon, Juan-Pablo Correa-Baena

**Affiliations:** † School of Materials Science and Engineering, 1372 Georgia Institute of Technology, Atlanta, Georgia 30332, United States; ‡ School of Chemistry and Biochemistry, Georgia Institute of Technology, Atlanta, Georgia 30332, United States; § Department of Physics and Center for Functional Materials, 8676 Wake Forest University, Winston–Salem, North Carolina 27109, United States; ∥ Institut Courtois, 5622 Université de Montréal, 1375 Avenue Thérèse-Lavoie-Roux, Montréal, Québec H2 V 0B3, Canada; ⊥ Department of Physics, 14770 City College of New York, New York, New York 10031, United States

**Keywords:** halide perovskites, polaritons, excitons, exciton-polariton condensation

## Abstract

Two-dimensional metal halide phases, commonly known as 2D perovskites, have emerged as promising materials for exciton-polaritons, particularly for polariton condensation. This process entails the spontaneous accumulation of population in the polariton ground state and relies on efficient energy relaxation. In this class of materials, this relaxation is mediated by exciton reservoir emission, which pumps polariton states through radiative pumping. To achieve strong light–matter coupling and sustain a high polariton density, the material must possess excitations with large oscillator strength and high exciton binding energy. While 2D perovskites exhibit these desirable characteristics, there are no reports of room-temperature polariton condensation and only one successful demonstration at cryogenic temperatures. In this work, we systematically explore the role of energy alignment between the exciton reservoir emission and the lower polariton branch in populating the polariton ground state via radiative pumping. Through cavity detuning, we shift the lower polariton energy minimum to overlap with the emission of the exciton reservoir at different energies. We identify that the multiple radiative pathways of 2D perovskites lead to inefficient radiative pumping of the lower polariton branch at the lowest-energy state, ultimately posing challenges for polariton condensation in this class of materials.

## Graphical abstract

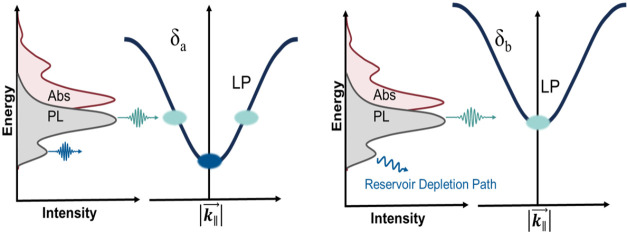

## Introduction

Microcavity exciton-polaritons are hybrid part-light part-matter quasiparticles that result from near-resonant, nondissipative energy exchange between excitons and modes of a confined electromagnetic field, in a regime known as strong light–matter coupling. In this regime, the energetics of the system can no longer be described by distinct light and matter excitations but instead by hybrid quasiparticles with featured properties from both: the upper and lower exciton-polaritons.^1–3^ Due to their mixed light–matter nature, polaritons inherit a low effective mass from light, and notable interactions from their matter constituent. These hybrid properties give them the ability to spontaneously form quantum phases with macroscopic coherence known as condensates.^2,4,5^ Polariton condensates are at the forefront of emergent classical and quantum technologies, acting as low-threshold lasers,^6^ optical logical gates^7,8^ and quantum bits for quantum information technology.^9^

Most studies of strong light–matter coupling have been performed in cavities containing traditional inorganic semiconductors such as GaAs,^10^ CdTe,^11^ ZnO,^12^ and GaN.^13^ Most of these group III–V semiconductors experience low exciton binding energies in their bulk form and hence require the formation of sophisticated quantum well structures and cryogenic temperatures to sustain excitons and undergo strong light–matter coupling.^14–16^ As an alternative to these materials, recent interest has turned to two-dimensional (2D) hybrid organic–inorganic metal halide phases, which form self-assembled quantum well structures hosting confined excitons, even at room temperature. 2D perovskites are promising candidates for polariton condensation due to their ease of thin film growth, high exciton binding energies (∼400 meV),^17–19^ high oscillator strengths,^17^ and tunable bandgap.^20^ However, despite these advantageous characteristics, reports of room-temperature polariton condensation in 2D perovskites remain elusive, with only one report at low temperatures existing in the literature.^21^ The unexpected complexity of achieving polariton condensation in these systems has driven significant efforts to understand the mechanisms governing exciton-polariton interactions in 2D perovskites.

The likelihood of a system to achieve polariton condensation depends on the efficiency with which polaritons accumulate at the lowest-energy and momentum state $(\left|\vec{k_{∥}\;}|=0\right)$ and reach a critical density, where their de Broglie wavelength exceeds the average interparticle spacing.^4,22,23^ Hence, the accumulation of polaritons at $\left|\vec{k_{∥}\;}\right|=0$ is fundamental for the formation of a condensate. In general, the relaxation of polaritons from higher- to lower-energy states along the polariton dispersion is mediated by many-body processes like parametric scattering^24–26^ and polariton-phonon scattering.^27–29^ However, recent research in the mechanisms by which exciton-polaritons exchange energy and momentum in 2D perovskite systems has shown evidence of a strong polariton bottleneck that hinders population relaxation to small $\left|\vec{k_{∥}\;}\right|$ .^30^ Such a bottleneck has been shown to disappear at low temperatures (below 60 K), where the emission spectrum of excitons in 2D perovskites becomes more defined.^30^ This observation demonstrates that the polariton relaxation mechanisms inevitably involve the ensemble of optically dark excitonic states, referred to as the exciton reservoir.

Furthermore, recent work by Deshmukh et al.^31^ has demonstrated that the population transfer from the exciton reservoir to the lower polariton branch is driven by the direct exchange of photons between excitons and polariton states, a process known as radiative pumping.^28,30–33^ This suggests that, to achieve a macroscopic accumulation of polaritons at the lowest-energy state, a similar approach to that used in molecular dye cavities^33^ and J-aggregate systems^34^ can be applied to 2D perovskite systems. In this context, radiative pumping can be used to directly feed the lower polariton branch at $\left|\vec{k_{∥}\;}\right|=0$ , bypassing the scattering mechanisms required for polaritons to relax in energy along the polariton dispersion.^21^ Hence, to achieve a critical concentration of polaritons at $\left|\vec{k_{∥}\;}\right|=0$ , it is fundamental to understand the radiative pumping processes taking place in the 2D perovskite microcavity system.

In this work, we designed microcavities with varying detuning, defined as the energy difference between the microcavity mode at normal incidence and the exciton absorption. This approach allowed us to shift the energy of the lower polariton and control its overlap with the emission maxima of the exciton reservoir while tracking the energy dispersion of the photoluminescence (PL) from the lower polariton branch. This provides us insights into the efficiency with which the exciton reservoir radiatively feeds the lower polariton. By performing this experiment as a function of temperature, we identify that the population distribution of the lower polariton depends on the spectral structure of the exciton reservoir emission. Similarly, we observe that the efficiency of radiative pumping depends on the nonresonant pumping fluence, which impacts the spectral shape of the emission of the exciton reservoir. Finally, we conclude that the multiple radiative pathways from the exciton reservoir decrease the effectiveness of radiative pumping in these systems at low temperatures, by allowing radiative recombination of the exciton reservoir at energies that do not directly populate the polariton ground state. This presents a major barrier to achieving polariton condensation.

## Results and Discussion

One of the most appealing aspects of 2D perovskites for polaritonics is their narrow, single-peak absorption and emission at room temperature (Figure S1a). However, as shown in Figure S1b, the low-temperature absorption spectrum reveals three excitonic features, while the emission spectrum exhibits two distinct emission peaks: a high-energy emission, referred to as PL1, and a low-energy emission, referred to as PL2. The origin of these spectral features remains debated, with explanations ranging from multiple distinct exciton polarons to vibronic progressions.^35–39^ Although the detailed nature of this fine spectral structure is beyond the scope of this work, the presence of these excited states shapes the complex photophysical landscape in this material that plays a key role in the strong light–matter coupling regime.^40^

To gain further understanding about the influence of the low-temperature fine excitonic structure in the distribution of the lower polariton population, we fabricated microcavities following the general structure depicted in Figure 1a. This structure comprises a 21 bilayer DBR (with a stopband centered at 520 nm), a 2D perovskite ((PEA)_2_PbI_4_) layer (Figure S2), an organic spacer layer made of poly­(methyl methacrylate) (PMMA), and a thin-top silver mirror. The low-temperature (5 K) absorption and photoluminescence spectra of the 2D perovskite bare film are shown in the left inset of Figure 1b, along with the energy dispersion measurement in reflectance of one of the microcavities fabricated (Figure 1b, right). The microcavity dispersion reveals four branches, with local reflectance minima seen in dark blue, corresponding to an upper polariton branch, a lower polariton branch, and two middle polaritons, the Rabi Splittings corresponding to these excitonic states are Ω_a_ ≃ 80, Ω_b_ ≃ 80, and Ω_c_ ≃ 90 meV, respectively (see Figure S3 and Table S1). These multiple polariton states observed at low temperatures correlate with the fine spectral structure of the neat film, shown in blue in the inset of Figure 1b. The multiple polariton branches result from the coupling between the microcavity photon mode and the different excitonic features in (PEA)_2_PbI_4_, which we observe in the absorption spectra (right, Figure 1b) and labeled as *X*_A_, *X*_B_, and *X*_c_.^35,41,42^ The energy dispersion of the microcavity agrees with the eigenstates of a Hamiltonian in which three excitons couple with a single microcavity mode. The numerical details of this Hamiltonian are provided in our previous work.^40^

**Figure 1:**
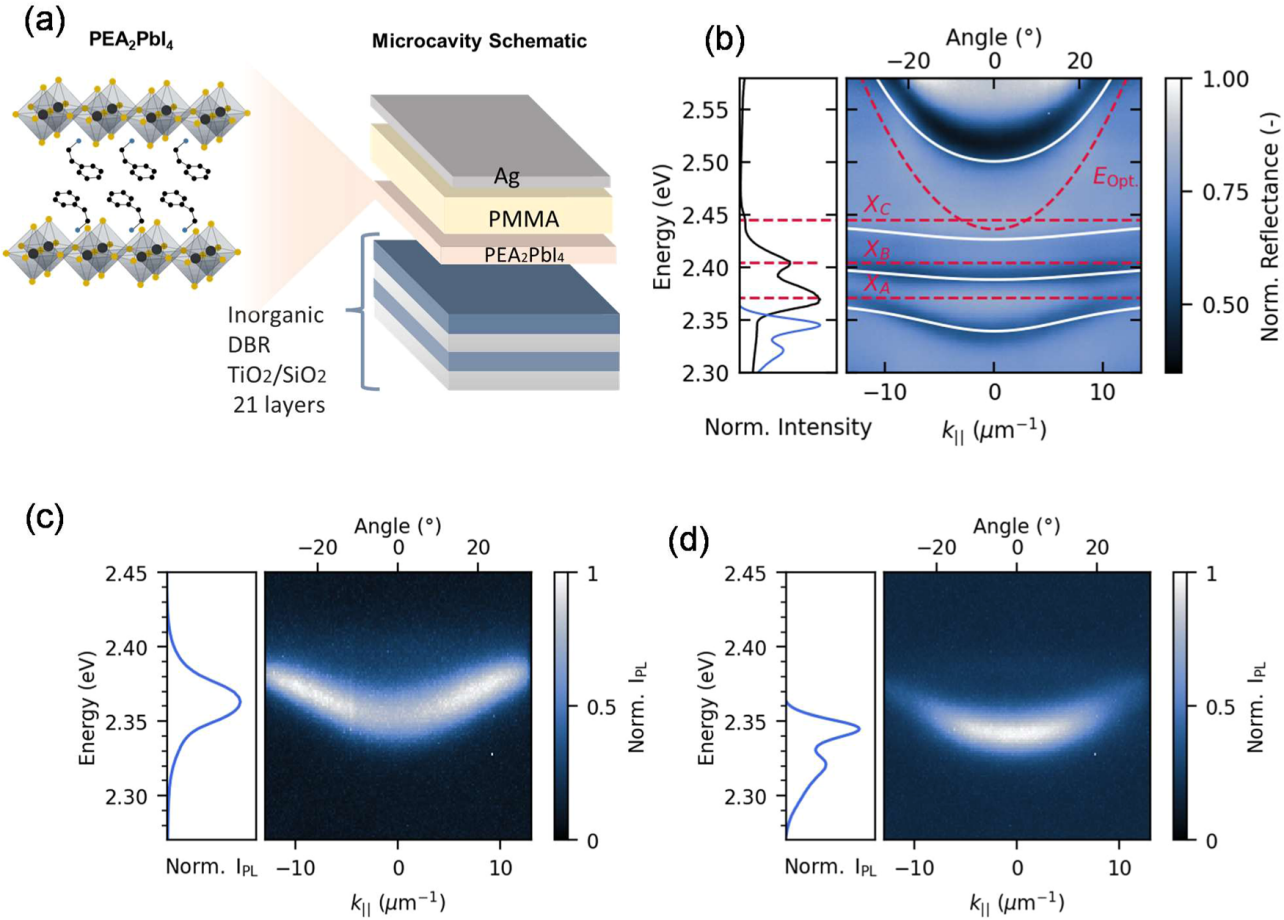
(a) Schematic of the (PEA)_2_PbI_4_ 2D perovskite microcavity, produced with a TiO_2_/SiO_2_ distributed Bragg reflector, a (PEA)_2_PbI_4_ layer, a poly­(methyl methacrylate) (PMMA) spacer film, and a Ag layer serving as a semitransparent top mirror. (b) Absorption and photoluminescence of the neat (PEA)_2_PbI_4_ film (left). The energy dispersion measured at 5 K with Fourier microscopy including the expected cavity mode and distinct exciton energies (red dashed line) as well as the simulated polariton modes (right). (c) 200 K *k⃗*-space photoluminescence dispersion showing accumulations of PL intensity at higher $\left|\vec{k_{∥}\;}\right|$ . (d) 5 K photoluminescence dispersion showing accumulation around $\left|\vec{k_{∥}\;}\right|=0$ .

The relationship between the emission from the exciton reservoir and the population distribution of the lower polariton can be directly visualized by measuring the photoluminescence energy dispersion of the microcavity at a series of temperatures. Figure 1c shows the PL dispersion at 200 K, where the increased PL intensity at larger in-plane wavevectors indicates radiative pumping, as it suggests that a greater population of polaritons is concentrated at the energy overlap between the exciton reservoir emission (inset) and the lower polariton dispersion. This is characteristic of polaritons populated via radiative pumping, followed by inefficient relaxation toward $\left|\vec{k_{∥}\;}\right|=0$ , in agreement with previous reports.^21,30^ At 5 K (Figure 1d), the apparent bottleneck at larger in-plane wavevectors disappears, and the lower polariton branch gets populated at smaller $\left|\vec{k_{∥}\;}\right|$ states. As discussed by Laitz et al.,^30^ the disappearance of the apparent polariton bottleneck at lower temperatures is not a consequence of increased scattering events down the polariton dispersion, but rather a result of radiative pumping from an exciton reservoir whose emission spectra is more defined and red-shifted at lower temperatures. This is evident from the inset of Figure 1d, where the steady-state PL of the uncoupled perovskite film is shown to radiate at lower energies, directly feeding polariton states around $\left|\vec{k_{∥}\;}\right|=0$ . Note that the lower polariton emission red-shifts at lower temperatures; this is a result of the temperature dependence of the band gap of phenethylammonium lead iodide (PEA)_2_PbI_4_, as shown in Figure S5 and previously rationalized in the literature as being caused by an anomalous temperature expansion coefficient in lead-based perovskites.^43^

To study the effect of energy alignment between the lower polariton mode minimum and the exciton reservoir emission, we fabricated cavities with theoretical quality factors (*Q*) in the order of 70, according to a transfer matrix simulation included in Figure S4, and different detuning (δ_1_ ≈ 80 and δ_2_ ≈ 40 meV, calculated with respect to x_B_ see Figure S3 a and c) achieved by varying the thickness of the PMMA layer from 134 to141 nm, respectively, and measured their photoluminescence energy dispersion at both low and high pumping fluences (Figure 2). First, we detuned the cavity mode by δ_1_ ≈ 80 meV to align the emission maxima of the neat perovskite film with the lower polariton mode minimum at $\left|\vec{k_{∥}\;}\right|=0$ . The fluence-dependent PL energy dispersion of this microcavity is displayed in Figure 2a,b, where the lower polariton branch energy aligns with the higher-energy PL peak (PL1). As the excitation fluence increases, the PL of the lower polariton branch accumulates towards $\left|\vec{k_{∥}\;}\right|=0$ , as shown by the increasing PL intensity at lower in-plane wavevectors. However, despite the high pumping fluence assessed, no condensation was observed.

**Figure 2:**
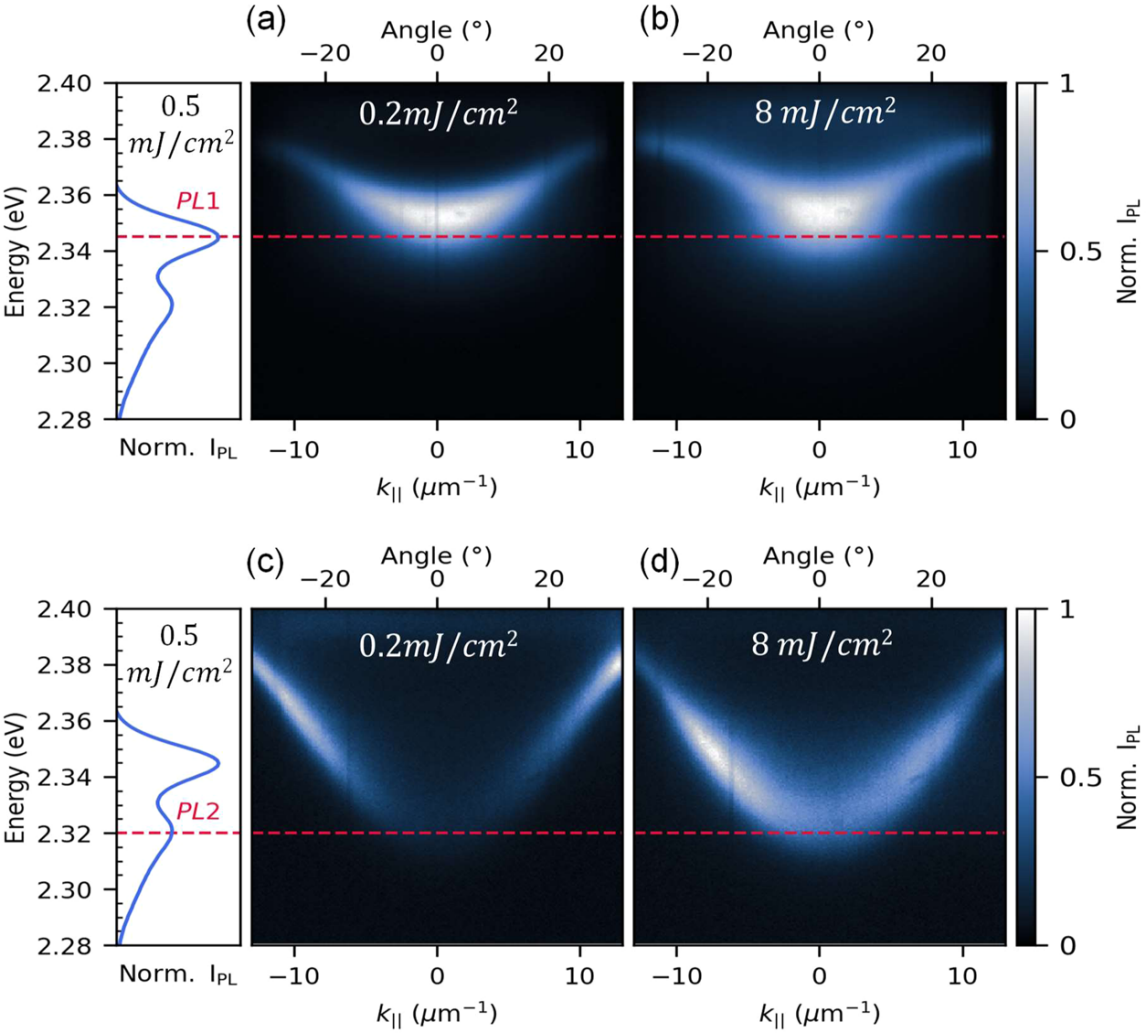
Photoluminescence energy dispersion, measured through Fourier microscopy, of a (PEA)_2_PbI_4_ microcavity with a detuning that maximizes the energy overlap between (a, b) the lower polariton and PL1 at a pumping fluence of 0.2 and 8 mJ/cm^2^, respectively. (c, d) The lower polariton and PL2 of the material at a pumping fluence of 0.2 and 8 mJ/cm^2^, respectively.

Similarly, we studied another cavity, detuned by δ_2_ ≈ 40 meV, in which the lower polariton mode matches the lower-energy emission of the exciton reservoir (PL2) at $\left|\vec{k_{∥}\;}\right|=0$ (Figure 2c,d). At low fluences, we observe that PL1 dominates the radiative pumping of the lower polariton, as evidenced by the higher emission intensity at larger in-plane wavevectors. In contrast, when the excitation fluence is increased, the maximum PL intensity shifts toward lower polariton states with smaller $\left|\vec{k_{∥}\;}\right|$ , indicating that their population grows with fluence. This suggests that the efficiency with which the incoherent PL2 emission populates the lower polariton branch through radiative pumping increases with fluence. We note that there was no degradation of the samples in the fluence range studied, as seen in Figure S8.

Next, to investigate why polariton condensation does not occur under radiative pumping through PL1 when it is energetically aligned with the polariton ground state, we experimentally examine the emission pathway of PL2, which does not overlap energetically with the lower polariton. For this, we fabricated low-quality factor cavities (*Q*_low_ ≈ 15) with thin-top mirrors and measured their PL energy dispersion as a function of fluence (Figure 3a,d). We observe leakage below the lower polariton mode at the energy of PL2. This photoluminescence feature does not follow a parabolic trend within the $\left|\vec{k_{∥}\;}\right|$ values assessed, suggesting that this emission is not polaritonic.^44^ Figure 3b shows cuts at the energy of the lower polariton branch (in red) alongside the PL2 emission attributed to the neat film. As fluence increases, the photoluminescence intensity of the lower polariton branch grows at a lower rate than the excitonic emission; however, both emissions plateau after a pumping fluence of 3 mJ/cm^2^ is reached. The difference in the rate of increase between the two PL intensities (lower polariton at $\left|\vec{k_{∥}\;}\right|=0$ and PL2) suggests that the exciton reservoir predominantly scatters through PL2 as the fluence increases. Therefore, in cavities where the lower polariton mode is radiatively pumped by PL1, the PL2 emission acts as a depletion mechanism for the exciton reservoir, dissipating energy by radiating at a region that does not populate polaritons.

**Figure 3:**
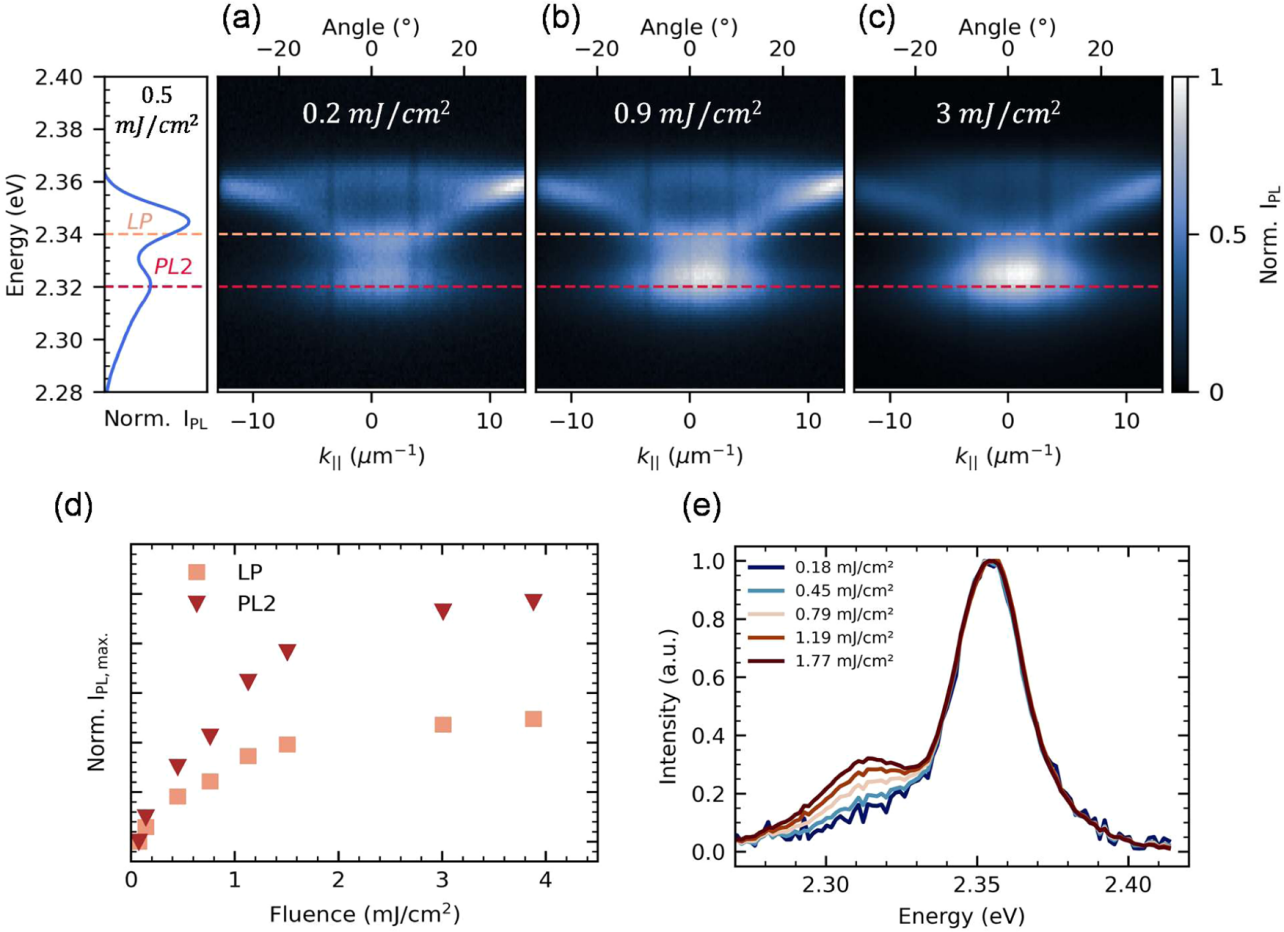
Fluence-dependent photoluminescence energy dispersion from thin-top mirror microcavities showing leakage from the exciton reservoir that increases with fluence, from 0.2 mJ/cm^2^ (a) to 0.9 mJ/cm^2^ (b) and 3 mJ/cm^2^ (c). The dashed horizontal cuts indicate the energies of the LP at $\left|\vec{k_{∥}\;}\right|=0$ (2.34 eV) and LP2 leakage (2.32 eV), respectively. (d) Maximum PL intensity at the lower polariton and exciton leakage energy. (e) PL from the 2D perovskite bare films as a function of fluence.

This fluence-dependent behavior of the exciton reservoir PL can be observed in the bare film PL collected at different pumping fluences, depicted in Figure 3e. As fluence rises from 0.18 to 1.77 mJ/cm^2^, the normalized intensity of the lower-energy peak (PL2) grows at a higher rate than that of the higher-energy peak (PL1). The fluence-dependent emission of this lower-energy feature in the PL had been reported previously^45^ as biexciton emission and was successfully used by Polimeno et al.,^21^ to feed the lower polariton mode through radiative pumping, ultimately driving the system into condensation. Figure S6 shows the power law-fitting of this data, we emphasize that the sublinear behavior observed in our measurements rules out supralinear processes such as biexciton emission or lasing. However, due to the complexity of the excitonic environment in these materials, with coexisting exciton families as evidenced in previous studies,^35,46,47^ the exact mechanisms leading to this emission remain an open question.

The sublinear photoluminescence intensity as a function of fluence of the lower polariton (LP) and excitonic emission in thin-top-mirror microcavities (Figure 3d) is indicative of nonlinear quenching mechanisms manifesting at higher excitation densities.^48,49^ To further understand such nonlinearities in the emission, and, more importantly, to rationalize the different intensity trends of the LP and PL2 peaks, we perform excitation correlation photoluminescence (ECPL) spectroscopy. In this experiment, the sample is photoexcited with two identical pump pulses with tunable time delay (τ) between them. Then, the photoluminescence measured from the sample will be composed of PL due to the excitation of each of the pulses and an additional cross-component (ΔPL), which arises only in the presence of nonlinear interactions between the photoexcited states. The relative fraction of this nonlinear component (ΔPL/PL) can be measured using lock-in methods, as described in the SI.

The ECPL signal can be interpreted as a precise indicator of the rate of change of the PL intensity with excitation density. In the absence of any interactions, the PL scales linearly with intensity, which results in a null ECPL response. A sublinear PL trend correlates with a negative ECPL response. This implies that the PL in the presence of both the pump pulses is lower than twice the PL from each of the individual pulses. The decay of the ECPL signal corresponds to the time it takes for the photogenerated population to return to a linear PL regime, if that exists for the sample under study.

With that brief introduction to ECPL, we proceed to investigate the PL nonlinearities of the thin-top-mirror cavity in which PL1 radiatively pumps the lower polariton. As noted earlier, the photoluminescence spectrum of this cavity has two features: emission from the lower polariton and PL2 exciton emission leaking from the cavity (see Figure 3). The spectrally integrated ECPL signal (Figure 4a) is negative and increases in magnitude with higher excitation fluence, which is consistent with the sublinear behavior of time-integrated LP and PL2 emission shown in Figure 3d. We also observe that the intensity of the ECPL signal reduces with increasing delay between the pump pulses, following a seemingly monoexponential decay time of about 30–40 ps. In our previous work,^40^ we reported very similar ECPL dynamics in a bare film of the 2D perovskite and a thick-top-mirror cavity with comparable detuning. In that work, given that the dynamics observed were much longer than the polariton lifetime and similar to or without the cavity, we consider them to be representative of the evolution of the population in the reservoir.

**Figure 4:**
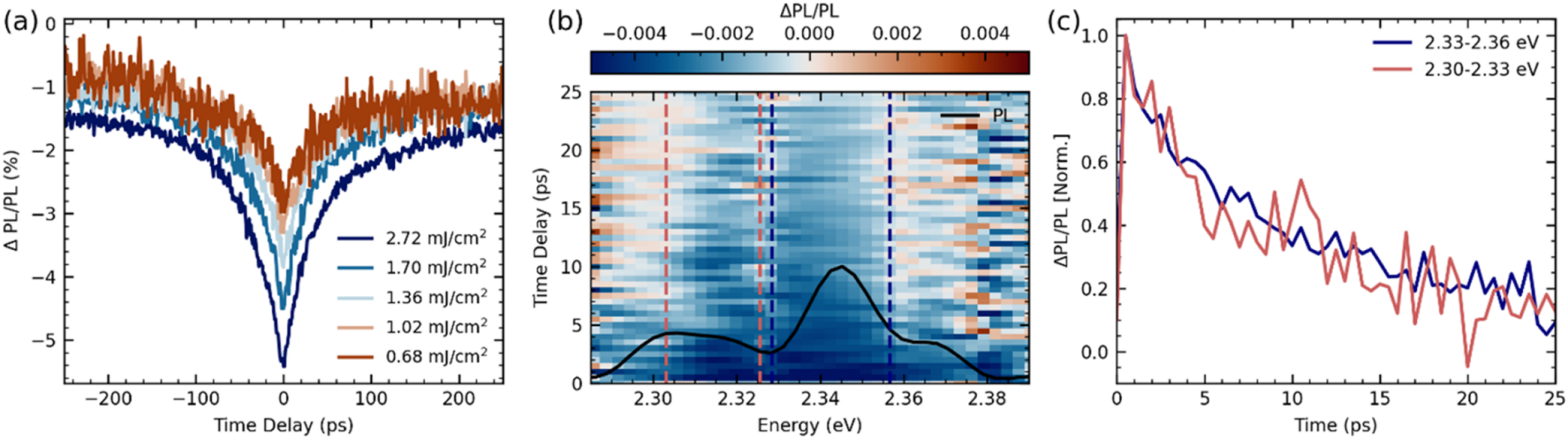
(a) Excitation correlation photoluminescence (ECPL) dynamics as the fractional change in the PL due to nonlinear interactions, plotted as a function of time delay and measured at different pump fluences. (b) Spectrally resolved map of ECPL dynamics taken with total pump fluence of 10 mJ/cm^2^. (c) Normalized ECPL dynamics integrated over the spectral region marked with the dotted line in (b). These correspond to the ECPL dynamics of the lower polariton emission and the emission from the PL2 peak.

The exciton–exciton annihilation rate and the monomolecular recombination rate of excitons govern the dynamics of the population in the reservoir. Here, the ECPL dynamics remain the same across all intensities. While the presence of an fluence-dependent negative ECPL response clearly suggests a nonlinear loss channel for the population, the dynamics indicate the overall relaxation of the exciton population in the tens of picosecond time scale. This can be rationalized by considering a bimolecular recombination rate, which at these exciton densities manifest in annihilation time scales that are orders of magnitude slower than the exciton lifetime. A more detailed explanation of this can be found in Supporting Information Note 1 and Figure S7. The observed decay rate of the ECPL is thus a measurement of the reduction in the exciton reservoir population due to the exciton lifetime within the sub-nanosecond timescale.

To further expand the investigation, we spectrally resolve the ECPL dynamics and Figure 4b shows the ECPL map as a function of the photon-energy and time delay. We note that if the ECPL response entirely arises due to the nonlinear interactions in the reservoir and given that a common reservoir feeds both the lower polariton and the PL2 state, we expect stationary ECPL intensity (ΔPL/PL) over the entire spectral range of the PL. Contrary to this, we observe that the negative nonlinear ECPL response is relatively higher for the lower polariton state in comparison to the PL2 state (see the SI). The larger nonlinearity at the the LP energy can be interpreted as enhanced interactions within the polariton lifetime, possibly mediated by cavity-enhanced excitonic interactions. This is consistent with the fluence dependence of the integrated PL, where the PL2 peak rises more rapidly than the LP peak with increasing excitation intensity (Figure 3d).

While the ECPL intensity is distinct at the LP and PL2 energies, it can be seen in the 2D map, as well as the integrated dynamics plotted in Figure 4c, that the ECPL evolution is identical for both the states, and entirely determined by the exciton lifetime, as seen in Figure 4c. We note the spectrally integrated dynamics in Figure 4a appear to have an additional long-living component, which is absent in the spectrally resolved dynamics in Figure 4c. This minor discrepancy arises due to sample inhomogeneities as those measurements have been performed on distinct sample spots. Nevertheless, the similarity in the ECPL dynamics at the LP and PL2 energies is evident. This supports our consideration that the reservoir is acting as a common source of population for both these states.

The ECPL dynamics highlight the presence of two distinct yet critical processes in the polariton dynamics. First, the exciton reservoir is continuously feeding both the lower polariton state and the PL2 state over its lifetime of over 50 ps. Hence, the reservoir that relaxes through the PL2 pathway does not participate in the radiative pumping process and leaks out of the microcavity, preventing an effective population of the LP state above the condensation threshold within the polariton lifetime. Second, additional nonlinearities may be present within the lower polariton state that manifest in sub-200 fs time scales, much faster than the time resolution of the current experiment. We have identified nonlinear scattering of polaritons close to $\left|\vec{k_{∥}\;}\right|=0$ within the first 100 fs in our recent report.^40^ The larger nonlinearity observed for the lower polariton in comparison with the excitonic leakage not only supports our earlier observation but also strengthens that this nonlinear scattering is detrimental to the population in the LP state. This further adds another critical factor that must be surmounted to facilitate the accumulation of sufficient population in the LP state for condensation.

The complex emission line shape of 2D perovskites poses a significant challenge for achieving efficient radiative pumping and polariton condensation in these systems. To reach polariton condensation, a critical density of polaritons must accumulate in the system’s lowest-energy state, which demands high-fluence, nonresonant pumping.^23^ However, the fluence-dependent behavior of the exciton reservoir emission introduces additional radiative recombination pathways that deplete the exciton reservoir and limit the available population to feed the lower polariton branch. The difficulty arises from the inability to radiatively pump the lower polariton at a narrow-energy region employing the exciton reservoir emission. As illustrated in Figure 5, the two emissions from the exciton reservoir yield a complex scenario for radiative pumping. Overcoming these limitations through materials design will be critical for developing 2D perovskite microcavities with polariton condensation.

**Figure 5:**
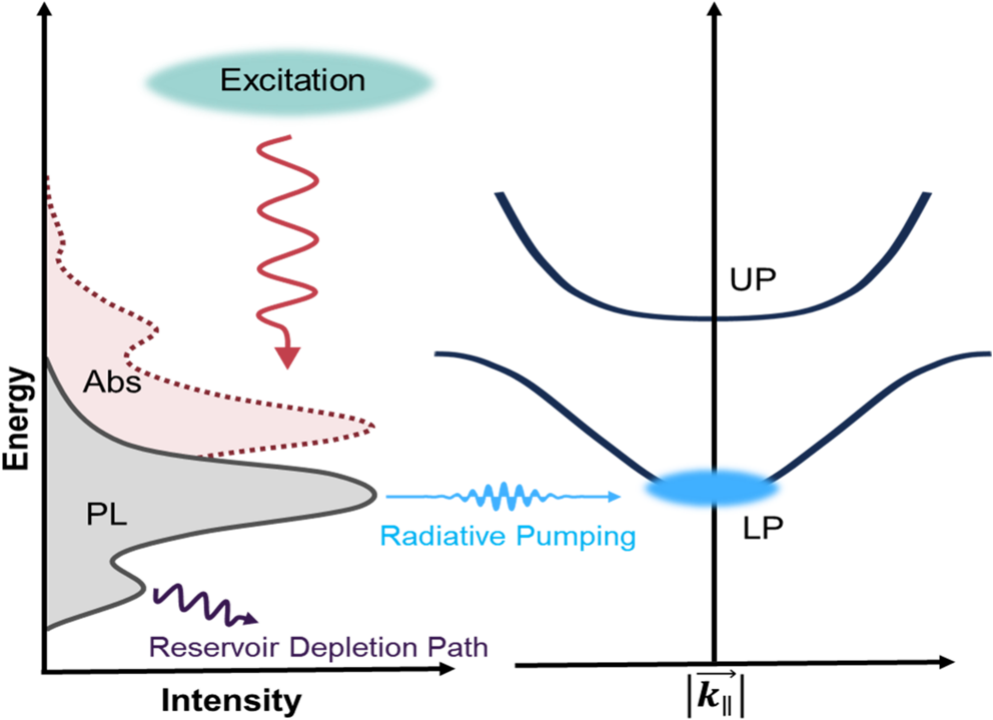
Exciton reservoir radiative recombination pathways. The two emission peaks lead to inefficient population of the lower polariton branch.

## Conclusions

The low-temperature fine structure of 2D perovskites presents significant challenges for polariton condensation, primarily due to its complex photophysics and multiple radiative pathways. Achieving a single, well-defined emission peak is critical for efficient radiative pumping and condensation. The presence of multiple emission features disperses the population of excited states and, when lacking energetic overlap with the lower polariton branch, provides an exciton reservoir depletion mechanism that limits the efficient feeding of polaritons into their lowest-energy state via radiative pumping. Our work highlights the need for advanced material design to control and simplify the emission line shape of 2D perovskites. By engineering the excitonic landscape and suppressing competing emission peaks, these emergent semiconductors can better support polariton condensation and unlock their potential for polariton-based technologies. Our findings advance the understanding of polariton dynamics in 2D perovskite systems and emphasize the critical role of fine-tuning material properties to achieve room-temperature polariton condensation.

## Experimental Methods

### Thin Film Preparation

The commercially available distributed Bragg reflectors (DBRs) with a stopband centered at 520 nm were cleaned in sequential ultrasonic baths of acetone and IPA for 15 min each, dried in nitrogen, and Uv-ozone-treated for 15 min. The perovskite precursor solutions were prepared by dissolving equimolar PbI_2_ (purity >99.99%) and phenethylammonium iodide (purity >99.99%) in *N,N*-dimethylformamide (purity >99.98%) at a 0.13 M concentration. The perovskite films were deposited by covering the 2.54 cm^2^ clean DBRs with 80 μL of precursor solution before spin coating them at 6000 rpm for 30 s with an acceleration of 6000 rpm/s. Immediately after, the perovskite films were thermally annealed for 10 min at 100 °C. The PMMA solution was prepared by dissolving 0.03 g of PMMA (Mw ∼15 ,000 g/mol) in 1 mL of toluene (purity > 99.98%) and allowed to dissolve for 24 h under constant agitation. The dissolved solution was then deposited by spin coating, 80 μL of solution was dropped on the finished perovskite layer and spin-coated in a 1-step process, at 6000 rpm, accelerated at 6000 rpm/s. The substrates were then thermally annealed for 5 min at 60 °C. Physical vapor deposition (PVD) was used for the top silver mirror, and silver pellets (purity >99.999%) were thermally evaporated at a rate of 0.5 Å/s to a final thickness of 42 nm.

### Thin Film Preparation and Characterization

The thin film materials characterization was performed at Georgia Tech in the Institute for Matter and Systems Materials Characterization Facilities. XRD measurements were done in ambient conditions on a Malvern PANalytical Empyrean with Bragg–Brentano geometry using a Cu–Kα source. The 2D perovskite films were deposited on soda lime glass. TEM images were taken using the Hitachi HT7700 TEM from films of 2D perovskites that were scrapped off and deposited on grids purchased from Ted Pella.

### Fourier Imaging

Using a home-built Fourier microscope, we imaged the energy dispersion of the reflectance and photoluminescence. The microscope employs a Zeiss LD EC Epiplan Neofluar 100X infinity-corrected objective (NA = 0.75), an Acton SpectraPro 300i spectrometer, and an Andor Newton EM camera. For reflectance and photoluminescence measurements, we use a ThorLabs SLS201L broadband light source and the output of an optical parametric amplifier (ORPHEUS, Light Conversion) at 470 nm pumped by a PHAROS laser (Model PH1-20-0200-02-10, Light Conversion), respectively.

### Excitation Correlation Photoluminescence Spectroscopy (ECPL)

In our setup, 1030 nm pulses are generated in a femtosecond laser system at a 10 kHz repetition rate (Pharos Model PH1-20-0200-02-12, Light Conversion). A portion of this output is used to feed a commercial optical parametric amplifier (Orpheus, Light Conversion), which generates our desired pulse energy of 2.75 eV (450 nm). The beam is then separated by a 50/50 beam splitter cube, and in order to control to delay between the two pulses, one beam is directed to a motorized linear stage (Thorlabs, LTS300). Each individual beam is modulated with a chopper at frequencies of 548 and 393 Hz, respectively. The beams are recombined in a parallel geometry and focused onto the sample with a microscope objective (20X Mitutoyo Plan Apo Infinity Corrected Long WD). The emitted photoluminescence (PL) is collected in reflection and directed to a high-speed photodetector (FEMTO, OE-200-SI-FS) using a dichroic mirror whose cutoff wavelength is 490 nm (Thorlabs, DMSP490). An additional long pass filter is placed to ensure complete removal of the pump, and then the signal is focused onto the photodetector. The photodetector is connected to a lock-in amplifier (Zurich Instruments, HF2LI), where the signal is demodulated at the sum frequency of the two beams (941 Hz). For the spectrally resolved ECPL measurements, a translating wedge-based interferometer (NIREOS, GEMINI) is placed into the path before the final lens which focuses the signal onto the detector. An interferogram is taken at each delay and the Fourier transform results in the spectrum. All measurements were taken with the sample at 15 K using a vibration-free coldfinger closed-cycle cryostat (Montana Instruments).

## Supplementary Material



## Notes

A previous version of this manuscript has been published as a preprint and can be found here: M.G-.D.; V.Q-.C.; E.R-.G.; K.A.K.; E.J.K.; C.A.R.P.; N.S.; C.S.; A.R.S.K.; V.M.; J-.P.C-.B. Multiple Emission Peaks Hinder Polariton Condensation in 2D Perovskite Microcavities. 2024 10.26434/chemrxiv-2024-3w9kv (accessed March 27, 2025).

## References

[1] Lidzey D. G. (1999). Room Temperature Polariton Emission from Strongly Coupled Organic
Semiconductor Microcavities. Phys. Rev. Lett..

[2] Jiang Z. (2022). Exciton-Polaritons and
Their Bose–Einstein Condensates in
Organic Semiconductor Microcavities. Adv. Mater..

[3] Weisbuch C., Nishioka M., Ishikawa A., Arakawa Y. (1992). Observation
of the
coupled exciton-photon mode splitting in a semiconductor quantum microcavity. Phys. Rev. Lett..

[4] Keeling J., Kéna-Cohen S. (2020). Bose-einstein condensation of exciton-polaritons
in
organic microcavities. Annu. Rev. Phys. Chem..

[5] Guillet T., Brimont C. (2016). Polariton condensates
at room temperature. C R Phys..

[6] Bajoni D. (2012). Polariton
lasers. Hybrid light–matter lasers without inversion. J. Phys. D Appl. Phys..

[7] Sannikov D. A. (2024). Room temperature, cascadable,
all-optical polariton universal gates. Nat.
Commun..

[8] Zasedatelev A. V. (2019). A room-temperature organic
polariton transistor. Nat. Photonics.

[9] Kavokin A. (2022). Polariton condensates
for classical and quantum computing. Nat. Rev.
Phys..

[10] Bajoni D. (2008). Polariton light-emitting diode in a GaAs-based microcavity. Phys. Rev. B:Condens. Matter Mater. Phys..

[11] Richard M., Kasprzak J., Romestain R., André R., Dang L. S. (2005). Spontaneous coherent phase transition
of polaritons
in CdTe microcavities. Phys. Rev. Lett..

[12] Li F. (2013). From excitonic to photonic
polariton condensate in a ZnO-based microcavity. Phys. Rev. Lett..

[13] Baumberg J. J. (2008). Spontaneous polarization
buildup in a Room-Temperature polariton
laser. Phys. Rev. Lett..

[14] Wurdack M. (2021). Motional narrowing,
ballistic transport, and trapping of room-temperature
exciton polaritons in an atomically-thin semiconductor. Nat. Commun..

[15] Calman E. V. (2018). Indirect excitons in
van der Waals heterostructures at room temperature. Nat. Commun..

[16] Duggan G., Ralph H. I. (1987). Exciton binding
energy in type-II GaAs-(Al,Ga)As quantum-well
heterostructures. Phys. Rev. B.

[17] Blancon J. C. (2018). Scaling law for excitons
in 2D perovskite quantum wells. Nat. Commun..

[18] Yaffe O. (2015). Excitons in ultrathin
organic-inorganic perovskite crystals. Phys.
Rev. B:Condens. Matter Mater. Phys..

[19] Tanaka K. (2005). Image charge effect
on two-dimensional excitons in an inorganic-organic
quantum-well crystal. Phys. Rev. B:Condens.
Matter Mater. Phys..

[20] Correa-Baena J.-P. (2017). Promises and challenges of perovskite
solar cells. Science (1979).

[21] Polimeno L. (2020). Observation of Two Thresholds
Leading to Polariton Condensation in
2D Hybrid Perovskites. Adv. Opt. Mater..

[22] Keeling J., Berloff N. G. (2011). Exciton–polariton
condensation. Contemp Phys..

[23] Kasprzak J. (2006). Bose–Einstein
condensation of exciton polaritons. Nature.

[24] Ciuti C., Schwendimann P., Quattropani A. (2003). Theory of
polariton parametric interactions
in semiconductor microcavities. Semicond. Sci.
Technol..

[25] Savvidis P. G. (2014). A practical
polariton laser. Nat. Photonics.

[26] Ciuti C., Schwendimann P., Quattropani A. (2001). Parametric luminescence of microcavity
polaritons. Phys. Rev. B.

[27] Tassone F., Yamamoto Y. (1999). Exciton-exciton scattering
dynamics in a semiconductor
microcavity and stimulated scattering into polaritons. Phys. Rev. B.

[28] Litinskaya M., Reineker P., Agranovich V. M. (2004). Fast polariton
relaxation in strongly
coupled organic microcavities. J. Lumin..

[29] Coles D. M. (2011). Vibrationally assisted
polariton-relaxation processes in strongly
coupled organic-semiconductor microcavities. Adv. Funct Mater..

[30] Laitz M. (2023). Uncovering temperature-dependent exciton-polariton relaxation mechanisms
in hybrid organic-inorganic perovskites. Nat.
Commun..

[31] Deshmukh P. (2023). Radiative pumping of
exciton-polaritons in 2D hybrid perovskites. Opt. Mater. Express.

[32] Mazza L., Fontanesi L., La Rocca G. C. (2009). Organic-based microcavities with
vibronic progressions: Photoluminescence. Phys.
Rev. B.

[33] Grant R. T. (2016). Efficient Radiative
Pumping of Polaritons in a Strongly Coupled Microcavity
by a Fluorescent Molecular Dye. Adv. Opt Mater..

[34] Lagoudakis K. G. (2013). Lasing through a strongly-coupled
mode by intra-cavity pumping. Opt. Express.

[35] Thouin F. (2019). Phonon coherences reveal the polaronic character of
excitons in two-dimensional
lead halide perovskites. Nat. Mater..

[36] Srimath
Kandada A. R., Silva C. (2020). Exciton Polarons in Two-Dimensional
Hybrid Metal-Halide Perovskites. J. Phys. Chem.
Lett..

[37] Neutzner S. (2018). Exciton-polaron spectral structures in two-dimensional hybrid lead-halide
perovskites. Phys. Rev. Mater..

[38] Straus D. B., Kagan C. R. (2018). Electrons, Excitons,
and Phonons in Two-Dimensional
Hybrid Perovskites: Connecting Structural, Optical, and Electronic
Properties. J. Phys. Chem. Lett..

[39] Dyksik M. (2024). Polaron Vibronic Progression
Shapes the Optical Response of 2D Perovskites. Adv. Sci..

[40] Quirós-Cordero, V. Competitive exciton and polariton scattering inhibits condensation in two-dimensional metal-halide-semiconductor microcavities. (2024).

[41] Straus D.
B., Kagan C. R. (2022). Photophysics
of Two-Dimensional Semiconducting Organic-Inorganic
Metal-Halide Perovskites. Annu. Rev. Phys. Chem..

[42] Neutzner S. (2018). Exciton-polaron spectral
structures in two-dimensional hybrid lead-halide
perovskites. Phys. Rev. Mater..

[43] Wang S. (2019). Temperature-Dependent
Band Gap in Two-Dimensional Perovskites: Thermal
Expansion Interaction and Electron-Phonon Interaction. J. Phys. Chem. Lett..

[44] Kavokin A. (2010). Exciton-polaritons
in microcavities: Recent discoveries and perspectives. Phys. Status Solidi B.

[45] Fang H. H. (2020). Band-Edge Exciton Fine Structure and Exciton Recombination Dynamics
in Single Crystals of Layered Hybrid Perovskites. Adv. Funct Mater..

[46] Thouin F., Cortecchia D., Petrozza A., Srimath Kandada A. R., Silva C. (2019). Enhanced screening
and spectral diversity in many-body elastic scattering
of excitons in two-dimensional hybrid metal-halide perovskites. Phys. Rev. Res..

[47] Straus D. B. (2016). Direct Observation of
Electron-Phonon Coupling and Slow Vibrational
Relaxation in Organic-Inorganic Hybrid Perovskites. J. Am. Chem. Soc..

[48] Rojas-Gatjens E. (2023). Resolving Nonlinear Recombination Dynamics in Semiconductors via
Ultrafast Excitation Correlation Spectroscopy: Photoluminescence versus
Photocurrent Detection. J. Phys. Chem. C.

[49] Silva C. (2001). Efficient exciton dissociation
via two-step photoexcitation in polymeric
semiconductors. Phys. Rev. B.

