# Supplementary material for: Multiple Emission Peaks Challenge Polariton Condensation in Phenethylammonium-Based 2D Perovskite Microcavities
Source: ACS Photonics. 2025 Apr 17;12(5):2423–31. doi: 10.1021/acsphotonics.4c02065 (PMC12100713; doi:10.1021/acsphotonics.4c02065)
Supplement: Supplementary file 1 [file ph4c02065_si_001.pdf]

Supplementary Information

Multiple Emission Peaks Challenge Polariton Condensation in Phenethylammonium-based 2D Perovskite Microcavities

Martin Gomez-Dominguez[1], Victoria Quirós-Cordero[1], Esteban Rojas-Gatjens[2], Katherine A Koch[3], Evan J Kumar[3], Carlo A.R Perini[1], Natalie Stingelin[1], Carlos Silva[1,2,4], Ajay Ram Srimath Kandada[3]*, Vinod Menon[5]*, Juan-Pablo Correa-Baena[1,2]*

[1]School of Materials Science and Engineering, Georgia Institute of Technology, Atlanta, GA, 30332, United States.

[2]School of Chemistry and Biochemistry, Georgia Institute of Technology, Atlanta, GA, 30332, United States.

[3]Department of Physics and Center for Functional Materials, Wake Forest University, Winston–Salem, North Carolina, 27109, United States.

[4] Institut Courtois, Université de Montréal, 1375 Avenue Thérèse-Lavoie-Roux, Montréal, Québec H2V 0B3, Canada.

[5]Department of Physics, City College of New York, New York, NY, 10031, United States.

*Corresponding authors: JPCB jpcorrea@gatech.edu, VM vmenon@ccny.cuny.edu, ARSK srimatar@wfu.edu

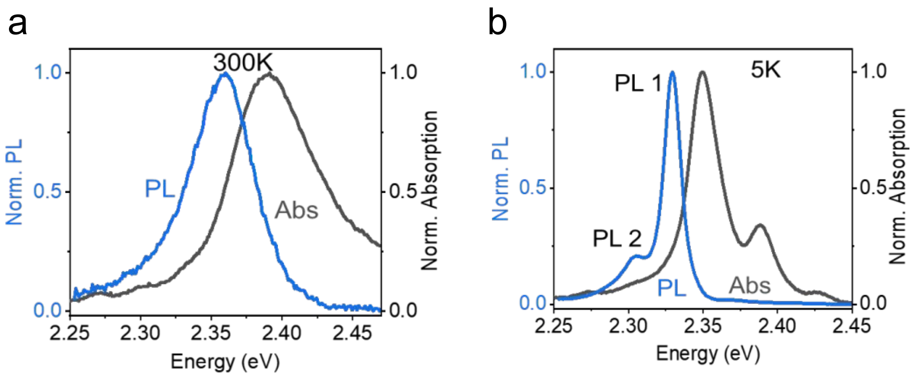

Figure S1. (a) Room temperature photoluminescence and absorption of PEA_2PbI_4. (b) Low temperature photoluminescence and absorption of PEA_2PbI_4 taken at 5K.

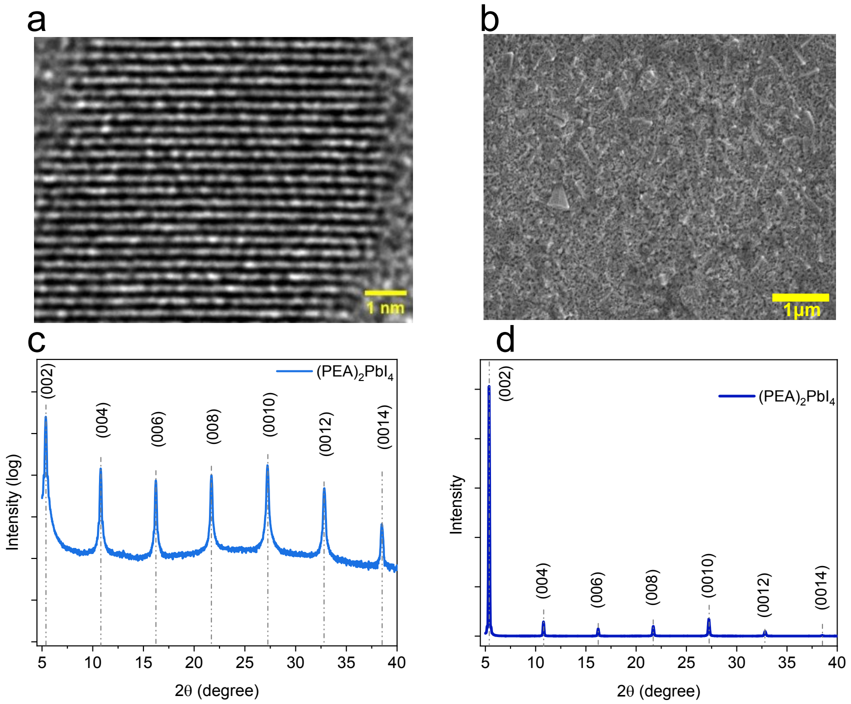

Figure S2. (a) Transmission and (b) scanning electron microscope images of a PEA_2PbI_4 thin film prepared for this study with the representative x-ray diffraction pattern. (c) X-ray diffraction plotted in log scale, showing preferential orientation along the <002> direction. (d) X-ray diffraction plotted in linear scale, showing low signal to noise ratio indicating strong crystallinity. The data shows the degree of order and quality of the materials we have prepared for this study.

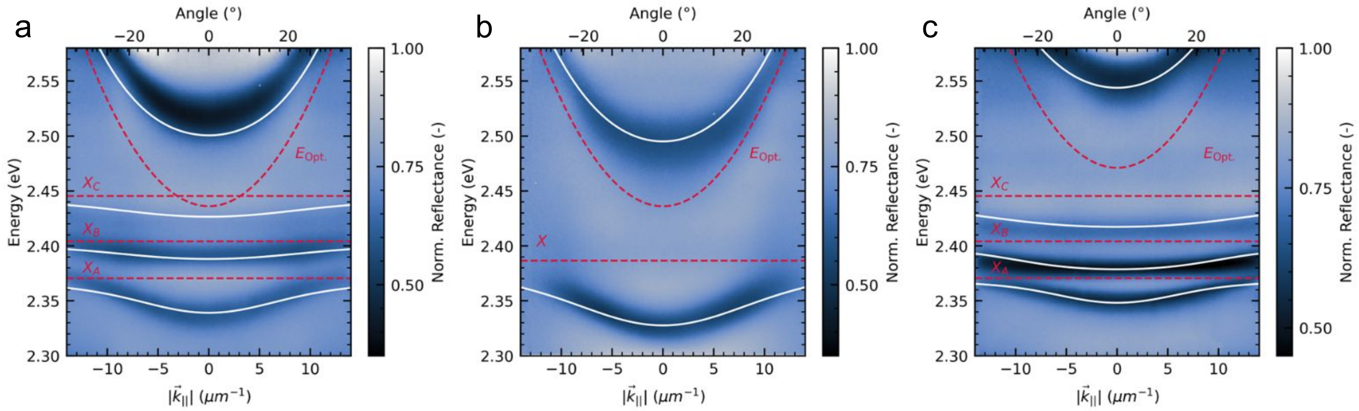

Figure S3. Experimental polariton dispersion in dark blue, matching the eigen states of a Hamiltonian (white solid line) in which three excitons couple with a single cavity mode (a) Thick mirror low temperature (5 K) microcavity (b) Room Temperature (298 K) thick mirror microcavity and (c) Thin mirror low temperature (5 K) microcavity. The Rabi Splitting extracted from the simulations are shown in Table S1**.**

**Microcavity**	$\mathbf{\Omega_a}$	$\mathbf{\Omega_b}$	$\mathbf{\Omega_c}$
Thick Mirror 5 K	80 meV	80 meV	90 meV
Thick Mirror 298 K	160 meV		
Thin Mirror 5 K	50 meV	85 meV	150 meV

Table S1. Rabi Splitting of microcavities at different temperatures, extracted from the simulations shown in Figure S3. Note that at room temperature, only one excited state is present, as observed in the absorption spectrum of Figure S1

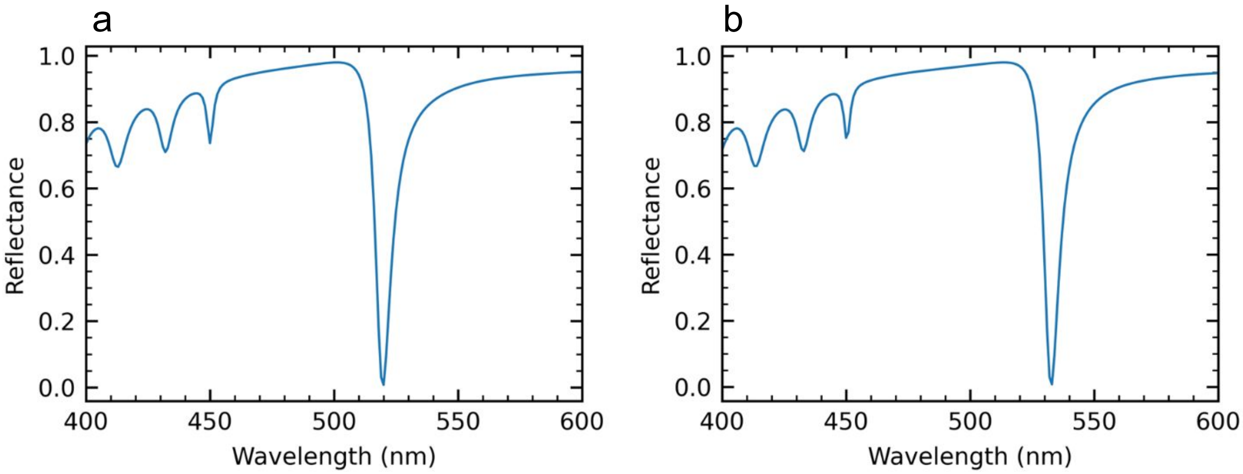

Figure S4. Normal incidence transfer matrix simulation for estimation of quality factors at (a) Detuning 1 (b) Detuning 2.

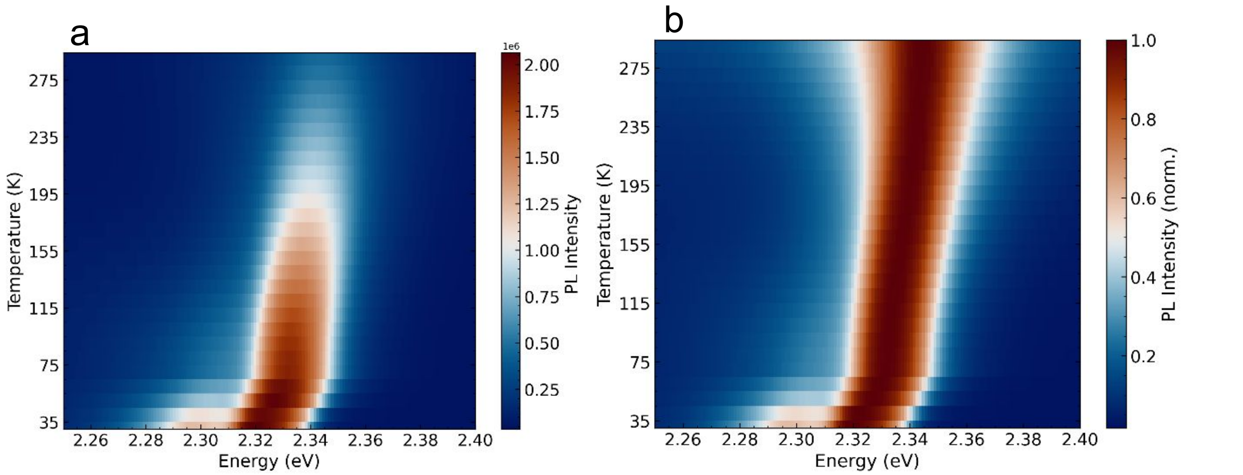

Figure S5. Temperature-dependent PL intensity maps of a $(PEA)_2PbI_4$ thin film, plotted with two normalization methods: (a) global normalization to visualize the overall changes in emission with temperature, and (b) local normalization at each temperature to better highlight the evolution of spectral shape and peak positions.

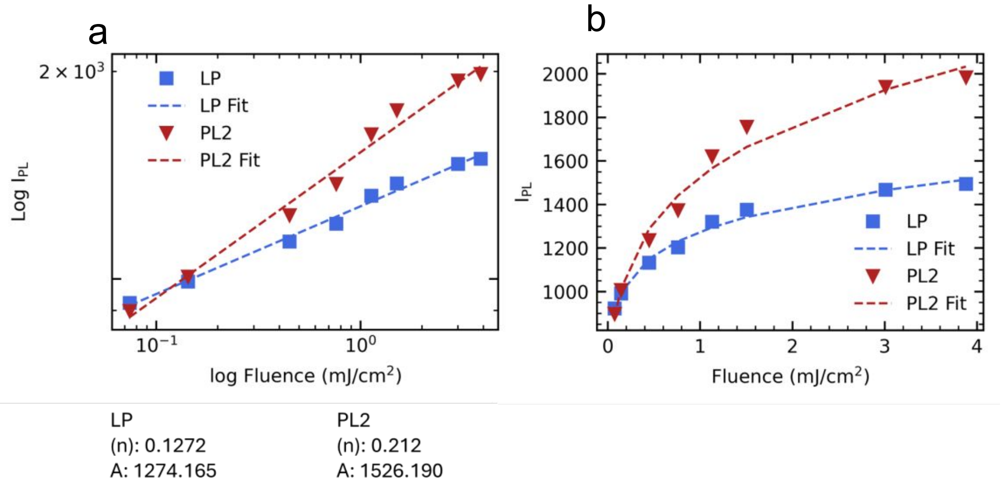

Figure S6. Power law fits of photoluminescence intensity as a function of fluence for the lower polariton (LP) and PL2 peaks. (a) Log-log plot highlighting the power law dependence of each emission feature where (n) is the power-law exponent and A is the y intercept. (b) Linear scale plot showing the fluence-dependent emission of the PL2 peak compared to the LP. Squares and triangles represent the experimental data, and dashed lines represent the power law fits.

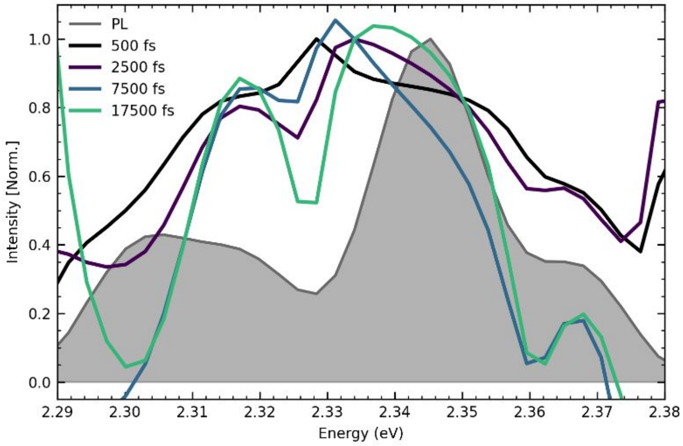

Figure S7: Normalized ECPL spectra at different time-delays plotted along with the PL spectrum. The relative ECPL intensity is higher at the LP energy than at the PL2 energy at all delays. Measurement noise is more apparent in the spectra measured at longer delays.

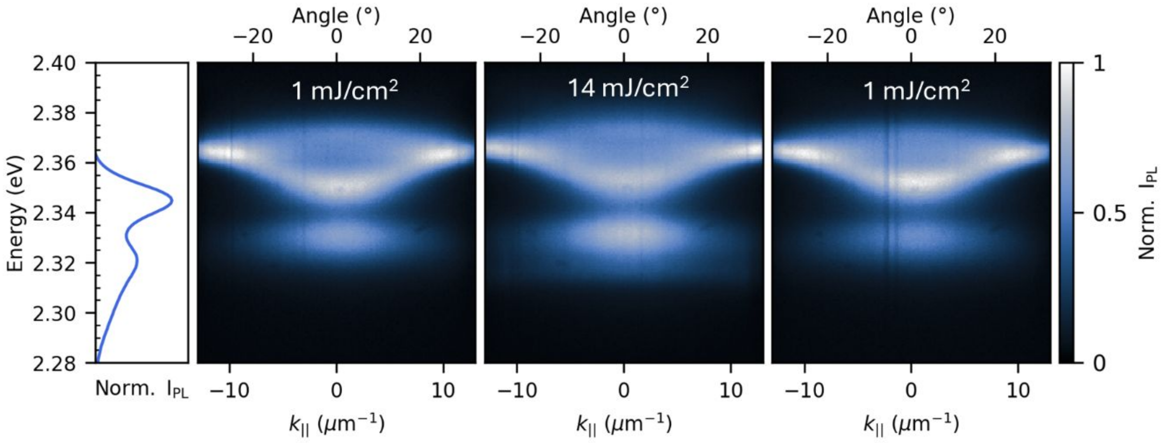

Figure S8: Emission spectra of the sample pumped sequentially at 1 mJ/cm², 14 mJ/cm², and returned to 1 mJ/cm². The consistency in intensity, shape, and energy of the emission of the lower polariton across all three conditions demonstrates that the sample does not undergo degradation at fluences up to 14 mJ/cm².

Supplementary Information Note S1:

In a generic scenario of monomolecular exciton recombination (γ) leading to the photoluminescence and bimolecular (β) quenching process, the population dynamics can be described as

$$\frac{dn_{ex}}{dt} = -\gamma n_{ex} - \beta n_{ex}^2$$

The intensity of the ECPL can be analytically written as:

$$I_{NPL} \approx \ln\left(1 - \frac{\alpha^2 \exp(-\gamma\tau)}{(1+\alpha)^2}\right),$$

Where $\alpha = \frac{n_0 \beta}{\gamma}$. Under the approximation of a very slow bimolecular recombination rate, which implies $\alpha \ll 1$, the above equation approximates a simple monoexponential decay with the decay rate given the monomolecular recombination rate.

$$I_{NPL} \propto - \frac{\alpha^2 \exp(-\gamma\tau)}{(1+\alpha)^2}$$

Supplementary Information Note S2:

Fluence Estimation:

It is important to note that the fluences reported in the manuscript are over estimations of the real fluence the sample experiences. The cavity is pumped non-resonantly and to account for the reflection losses, we estimated the cavity reflects about 40% of the input pump beam power. This loss was taken into account when determining the fluence.
